# Potential of *Aedes albopictus* and *Aedes aegypti* (Diptera: Culicidae) to transmit yellow fever virus in urban areas in Central Africa

**DOI:** 10.1080/22221751.2019.1688097

**Published:** 2019-11-12

**Authors:** Basile Kamgang, Marie Vazeille, Aurélie P. Yougang, Armel N. Tedjou, Theodel A. Wilson-Bahun, Laurence Mousson, Charles S. Wondji, Anna-Bella Failloux

**Affiliations:** a Department of Medical Entomology, Centre for Research in Infectious Diseases, Yaoundé, Cameroon; b Department of Virology, Institut Pasteur, Unit of Arboviruses and Insect Vectors, Paris, France; c Department of Animal Biology, Faculty of Sciences, University of Yaoundé I, Yaoundé, Cameroon; d Faculty of Science and Technology, Marien Ngouabi University, Brazzaville, Congo; e Vector Biology Department, Liverpool School of Tropical Medicine, Liverpool, UK

**Keywords:** *Aedes aegypti*, *Aedes albopictus*, yellow fever virus, vector competence, Central Africa

## Abstract

Yellow Fever (YF) remains a major public health issue in Sub-Saharan Africa and South America, despite the availability of an effective vaccine. In Africa, most YF outbreaks are reported in West Africa. However, urban outbreaks occurred in 2016 in both Angola and the Democratic Republic of Congo (DRC), and imported cases were reported in Chinese workers coming back from Africa. In Central Africa, Cameroon and the Republic of Congo host a high proportion of non-vaccinated populations increasing the risk of urban outbreaks. The main vector is *Aedes aegypti* and possibly, *Aedes albopictus,* both being anthropophilic and domestic mosquitoes. Here, we provide evidence that both *Ae. aegypti* and *Ae. albopictus* in Cameroon and the Republic of Congo are able to transmit Yellow fever virus (YFV) with higher rates of infection, dissemination, and transmission for *Ae. aegypti*. We conclude that the potential of both *Aedes* species to transmit YFV could increase the risk of urban YF transmission and urge public health authorities to intensify their efforts to control domestic vectors, and extend vaccine coverage to prevent major YFV outbreak.

## Background

Yellow fever (YF) is a mosquito borne viral disease endemic in South America and Sub-Saharan African countries. Clinical signs vary from a fever with aches and pains to severe liver disease with bleeding and yellowing skin (jaundice), for which there is no specific treatment. Despite the availability of an effective vaccine, which can offer a lifelong immunity, numerous cases of YF are still being reported. Indeed, a modelling study based on African data sources estimated that the burden of YF during 2013 was 84 000–170 000 severe cases and 29 000–60 000 deaths [1]. Yellow fever virus (YFV, *Flavivirus*, *Flaviviridae*) is transmitted to humans and non-human primates mainly by bites of infected mosquitoes belonging to *Aedes* and *Haemagogus* genera*.* This virus primarily circulates in the forest between non-human primates and sylvatic *Aedes* spp*.* mosquitoes (e.g. *Ae. africanus*) in Africa and *Haemagogus* spp*.* in South America [2]. Nevertheless, YF can spread widely in urban environments when transmitted from human to human by the anthropophilic mosquitoes, *Aedes aegypti* [3] or potentially, *Aedes albopictus* [4,5]. Indeed, between 2015 and 2016 in Central Africa, major urban YF outbreaks occurred in Angola and Democratic Republic of Congo with 7,334 suspected cases, of which 962 have been confirmed, and 393 deaths [6]. *Aedes aegypti* was suspected as the main YFV vector involved during the Angola outbreak due to its high densities reported across the country [7]. On the other hand, recent studies on entomological surveillance in Central Africa particularly in Cameroon [8] and the Republic of Congo [9], where sporadic cases of YF were frequently reported, showed that *Ae. aegypti* is present in all urban environments while *Ae. albopictus* introduced in 2000s has a distribution limited under 6°N latitude. In sympatric areas, *Ae. albopictus* tends to be the most prevalent species by replacing the resident species *Ae. aegypti* [8–10]. In Cameroon, the first isolation of YFV was in 1990 during an outbreak with 180 cases, of which 125 fatalities [11]. The suspected mosquito vectors were *Ae. aegypti*, *Ae. furcifer*, and *Ae. luteocephalus*. From 2010 to 2016, 13,837 suspected cases of YF were reported of which 109 cases were confirmed with 66% mostly in rural areas [7]. The epidemiological importance of both vectors in urban YFV transmission in Central Africa has not been assessed precisely up to now. As the vector competence is one of the key parameters to assess the pathogen transmission, we undertook a study aimed at establishing the ability of *Ae. aegypti* and *Ae. albopictus* populations collected in different urban settings in Central Africa to transmit YFV strain isolated in West Africa.

## Material and methods

### Ethics statement

This study was approved by the Cameroonian national ethics committee for human health research N˚2017/05/911/CE/CNERSH/SP. Oral consent to inspect the potential breeding sites was obtained in the field in household or garage owners. The Institut Pasteur animal facility received accreditation from the French Ministry of Agriculture to perform experiments on live animals in compliance with the French and European regulations on care and protection of laboratory animals (EC Directive 2010/63, French Law 2013-118, 6 February 2013). All experiments were approved by the Ethics Committee and registered under the reference APAFIS6573-201606l412077987 v2.

### Mosquito sampling

Larvae and pupae were collected from August 2017 to April 2018 in several locations in Central Africa including the Republic of Congo (Brazzaville) and Cameroon (Yaoundé, Douala, Tibati and Bénoué National Park). Each of these locations has been previously described [8,9] except Bénoué National Park (8°20′N, 13°50′E); it is a biosphere reserve located in Northern part of Cameroon on the Bénoué River plain, at the foot of the Adamawa plateau. In Benoué park, *Aedes* larvae were collected across the park in tree holes (1), tin cans (15), used tires (2) and discarded chair (1). For other locations, mosquitoes were collected in peri-urban and downtown in a minimum of 20 containers per environment. Immature stages of *Aedes* were transported in the insectary and pooled together according to the city and raised until adults before morphological identification using criteria established by Jupp (1996) [12]. Adult mosquitoes were pooled together according to the location, species and reared at 28°±1°C under 12 h dark:12 h light cycle and 80% relative humidity. Eggs obtained (Table 1) were transported to the Institut Pasteur in Paris, reared to adult stage and used to challenge with YFV.

**Table 1.** Origin of Ae. aegypti and Ae. albopictus used for vector competence.

Location	Species	Generation
Yaoundé	*Ae. albopictus*	G2
Tibati	*Ae.albopictus*	G2
Douala	*Ae. albopictus*	G2
Brazzaville	*Ae. albopictus*	G5
Yaoundé	*Ae. aegypti*	G2
Bénoué Parc	*Ae. aegypti*	G4
Brazzaville	*Ae. aegypti*	G2
Douala	*Ae. aegypti*	G2

## Virus strain

YFV was isolated from a human case in Senegal in 1979 (YFV S79; accession number: MK060080) [13]. YFV S79 was passaged twice on newborn mice and two times on C6/36 cells. Viral stocks were produced on *Aedes albopictus* C6/36 cells.

### Challenge mosquitoes with YFV

For each population, six batches of 60 7–10 day-old females were challenged with an infectious blood meal containing 1.4 mL of washed rabbit erythrocytes and 700 μL of viral suspension. The blood meal was supplemented with adenosine 5’-triphosphate (ATP) as a phagostimulant at a final concentration of 1 mM and provided to mosquitoes at a titer of 10^7^ focus-forming unit (FFU)/mL using a Hemotek membrane feeding system (Hemotek Ltd, Blackburn, UK). Mosquitoes were allowed to feed for 20 min through a piece of pork intestine covering the base of a Hemotek feeder maintained at 37°C. Fully engorged females were transferred in cardboard containers and maintained with 10% sucrose under controlled conditions (28±1°C, relative humidity of 80%, light:dark cycle of 12h:12 h) for up to 21 days with mosquito analysed at 14 and 21 days post-infection (dpi). 21–32 mosquitoes were examined at each dpi.

## Infection, dissemination and transmission assays

For each mosquito examined, body (abdomen and thorax) and head were tested respectively for infection and dissemination rates at 14 and 21 dpi per population when the number permitted. For this, each part was ground individually in 300 μL of L15 medium (Invitrogen, CA, USA) supplemented with 2% fetal serum bovine (FBS), and centrifuged at 10,000×g for 5 min at +4°C. The supernatant was processed for viral titration. Saliva was collected from individual mosquitoes at 21 dpi using the forced salivation technique as described previously [14]. Briefly, mosquitoes were cool anesthetized, wings and legs of each mosquito were removed and the proboscis inserted into a tip of 20 µL containing 5 µL of FBS. After 30 min, FBS containing saliva was added to 45 µL of L15 medium for titration. Transmission rates were assessed only at 21 dpi based on previous studies demonstrated that higher transmission rates were reported at this time point [15].

Infection rate (IR) refers to the proportion of mosquitoes with infected body (i.e. abdomen and thorax) among tested mosquitoes. Disseminated infection rate (DIR) corresponds to the proportion of mosquitoes with infected head among the previously detected infected mosquitoes (i.e. virus positive abdomen/thorax). Transmission rate (TR) represents the proportion of mosquitoes with infectious saliva among mosquitoes with disseminated infection. Vector competence can be summarized by the transmission efficiency (TE) which was calculated as the proportion of mosquitoes with infectious saliva among all mosquitoes tested [16].

## Viral titration by focus forming assay

Samples were titrated by focus fluorescent assay on C6/36 *Ae*. *albopictus* cells [17]. Body, head and saliva suspensions were serially diluted in L15 medium supplemented with 2% of FBS and inoculated onto cells in 96-well plates. After an incubation of 5 days at 28°C, samples were fixed with 0.1 mL/well of formaldehyde 3.6% in phosphate buffer saline (PBS) during 20 min at room temperature. Then, plates were stained using antibodies specific to YFV (Bio-techne, Minneapolis, Minnesota, USA) as the primary antibody and conjugated Alexa Fluor 488 goat anti-mouse IgG as the second antibody (Life Technologies, California, USA). Titers were expressed as FFU/mL.

## Statistical analysis

All statistical analyses were performed with R software v 3.5.2 (R Core Team, Vienna, Austria). Qualitative variables were expressed as proportion and compared using Fisher’s exact test the RVAideMemoire package and quantitative variables by mean and compared using non-parametric test of Kruskal–Wallis because of non-normal distribution. *P-value* <0.05 was considered as statistically different.

## Results

### Infection and disseminated infection rates in *Ae. albopictus* and *Ae. aegypti*

**Figure 1.**
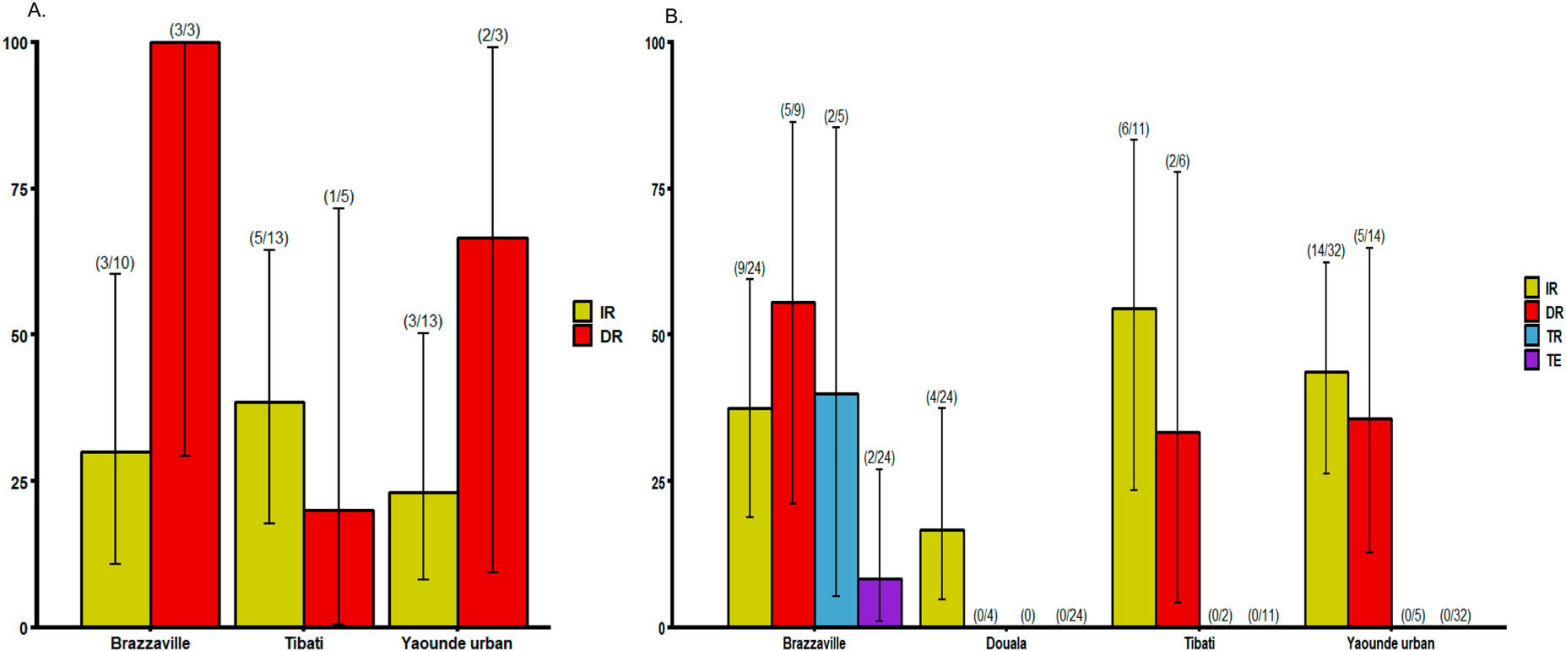
Infection, disseminated infection, transmission rates and transmission efficiency of *Ae. albopictus* from Central Africa to yellow fever virus. (A) Infection and disseminated infection rates at 14 days post-infection (dpi). (B) Infection, disseminated infection, transmission rates and transmission efficiency at 21 dpi. Error bars show the 95% confidence interval. In brackets, the number of mosquitoes examined. IR: the proportion of mosquitoes with infected body among engorged mosquitoes; DIR: the proportion of mosquitoes with infected head among mosquitoes with infected body; TR: the proportion of mosquitoes with infectious saliva among mosquitoes with infected head. TE: the proportion of mosquitoes with infectious saliva among all analysed ones.

**Figure 2.**
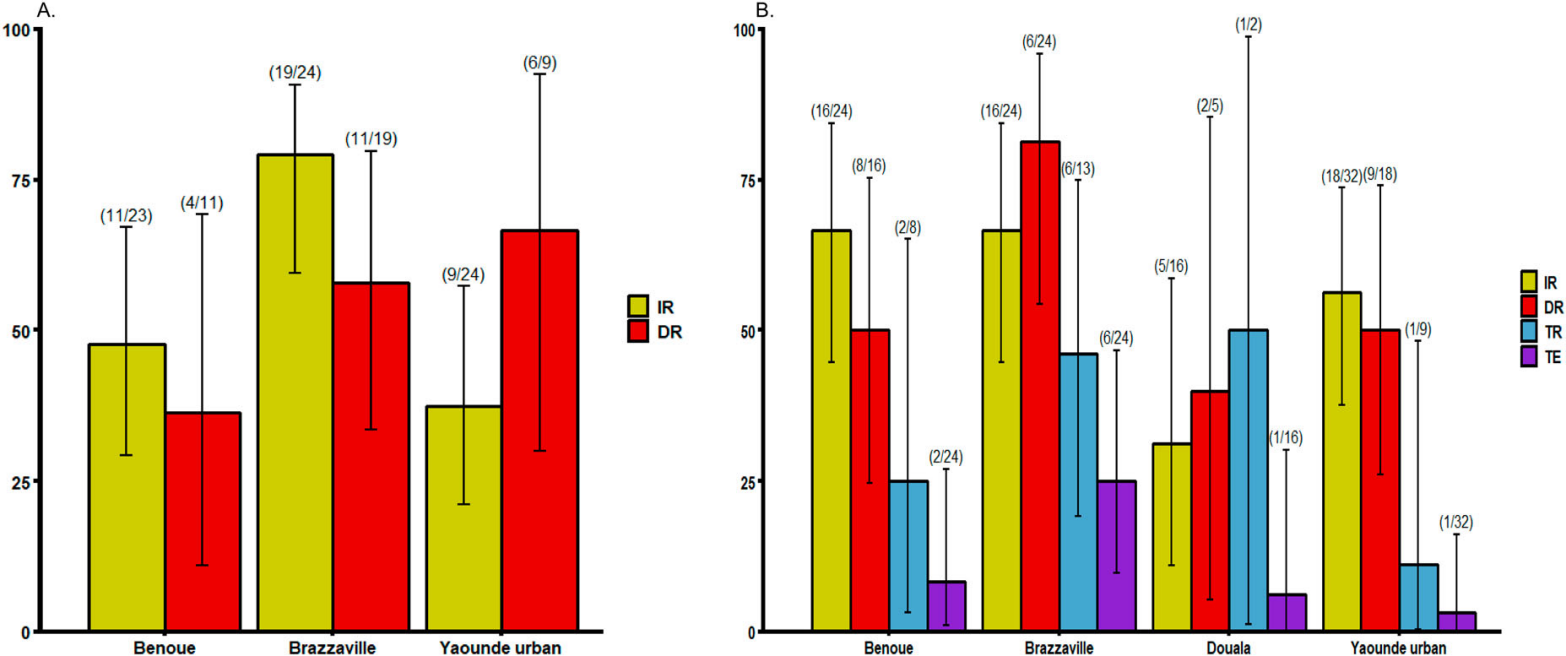
Infection, disseminated infection, transmission rates and transmission efficiency of *Ae. aegypti* from Central Africa to yellow fever virus. (A) Infection and disseminated infection rates at 14 days post-infection (dpi). (B) Infection, disseminated infection, transmission rates and transmission efficiency at 21 dpi*.* Error bars show the 95% confidence interval. In brackets, the number of mosquitoes examined. IR: the proportion of mosquitoes with infected body among engorged mosquitoes; DIR: the proportion of mosquitoes with infected head among mosquitoes with infected body; TR: the proportion of mosquitoes with infectious saliva among mosquitoes with infected head. TE: the proportion of mosquitoes with infectious saliva among all analysed ones.

Mosquitoes were analysed at two time points following the infectious blood meal when the number of mosquitoes was sufficient: 14 and 21 days post infection (dpi) (Figures 1 and 2). At 14 dpi, no significant difference of infection rate (IR) and disseminated infection rate (DIR) was found between *Ae. albopictus* populations, respectively *P *= 0.89 and *P *= 0.18 (Fisher’s exact test) (Figure 1A). Besides, in *Ae. aegypti,* IRs were significantly different (Fisher’s exact test: *P*=0.01) ranging from 37.5% in Yaoundé to 79.2% in Brazzaville (Figure 2A) while DIRs were not significantly different (Fisher’s exact test: *P *= 0.49). When considering all populations of same species, IRs for *Ae. aegypti* (mean = 54.9%) was significantly higher than *Ae. albopictus* (mean = 30.7%) (Fisher’s exact test: *P *= 0.02) while DIRs were not significantly different (*Ae. aegypti:* mean = 56.8% and *Ae. albopictus:* mean = 54.5%). At 21 dpi, IRs for *Ae. albopictus* ranged from 16.8% in Douala to 54.5% in Tibati and were not statistically different (Figure 1B; Fisher’s exact test: *P*=0.08). In *Ae. albopictus,* DIRs ranged from 0 for Douala population suggesting no dissemination to 55% in Brazzaville population. Meanwhile, for three populations where viral dissemination was reported, no statistical difference was found (Figure 1B; Fisher’s exact test: *P *= 0.34). In *Ae. aegypti*, IRs varied between 31.2% (Douala) and 66.7% (Bénoué, Brazzaville) and were not statistically different (Fisher’s exact test: *P *= 0.11). DIRs were not significantly different (Fisher’s exact test: *P *= 0.14) ranging from 40% (Douala) to 81.2% (Brazzaville) (Figure 2B). Overall, at 21 dpi, IRs were higher for *Ae. aegypti* (54.5%) than for *Ae. albopictus* (16.7%) (Fisher’s exact test: *P *= 0.005) while for DIRs, no significant difference was reported between both species (Fisher’s exact test: *P *> 0.5). When considering the two mosquito species from a same location, IRs and DIRs were not significantly different (Fisher’s exact test: *P > 0.05*) except for IRs of *Ae. aegypti* and *Ae. albopictus* from Brazzaville at 14 dpi (*P *= 0.013).

## Transmission rate and transmission efficiency

**Figure 3.**
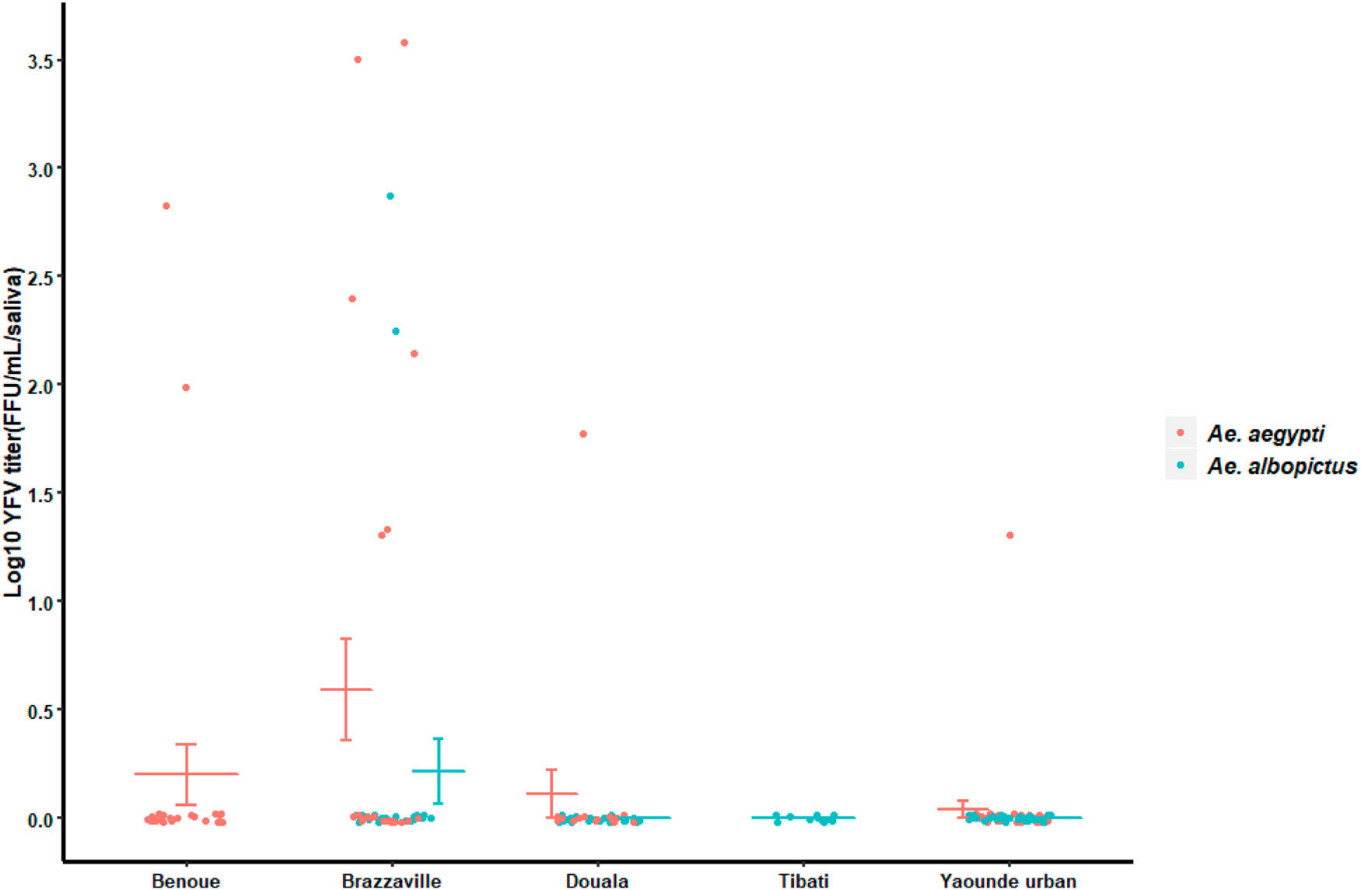
Yellow fever virus titers in saliva of *Ae. aegypti* and *Ae. albopictus* at 21 days post-infection. The bars indicate the confidence interval of the mean for viral load in each population.

Our analysis showed that YFV was able to replicate, disseminate and be excreted in saliva of both *Ae. albopictus* and *Ae. aegypti* (Figures 1B and 2B)*.* However, in *Ae. albopictus*, YFV was detected only in saliva of Brazzaville population. In contrast, in *Ae. aegypti,* YFV was found in saliva of all tested populations with transmission rate (TR) and transmission efficiency (TE) ranging from 11.1% (Yaoundé) to 50% (Douala) and 3.2% (Yaoundé) to 25% (Brazzaville) respectively. Collectively, *Ae. aegypti* exhibited a higher TE (10.4%) than *Ae. albopictus* populations (2.2%) (Fisher’s exact test: *P *= 0.03). In *Ae. aegypti,* viral titers varied significantly from Yaoundé population to Brazzaville population (Figure 2; Chi-squared = 7.91; df = 3; *P *= 0.04). In *Ae. albopictus* Brazzaville population, viral load in saliva was higher than in some *Ae. aegypti* populations (Figure 3).

## Discussion

Yellow fever virus is circulating in Central Africa where massive outbreaks have been reported recently in Angola and the Democratic Republic of Congo [6] in spite of the availability of an effective vaccine. In this study, we assessed the ability of *Ae. aegypti* and *Ae. albopictus* collected in different ecological settings in Cameroon and the Republic of Congo to transmit YFV isolated from a human case in Senegal. Our analysis showed that YFV was able to replicate, disseminate and be excreted in saliva of both *Ae. aegypti* and *Ae. albopictus* from Central Africa at 21 dpi*.* High levels of infection and disseminated infection rates were reported in both species from different locations. YFV was only detected in saliva of a single population of *Ae. albopictus* from Brazzaville (Congo) at 21 dpi with a transmission rate comparable to that found for *Ae. albopictus* populations from South France and Morocco [4,18], suggesting a low potential of this species to sustain an active viral transmission. Furthermore, YFV was found at 21 dpi in saliva of all populations of *Ae. aegypti* from different ecological settings, indicating a higher epidemiological risk related to this mosquito in urban areas. Interestingly, transmission rate reported in *Ae. aegypti* populations was similar to those reported in previous studies undertaken in Carbo Verde [19], Brazil [20] and Guadeloupe [15] using the same YFV strain (Senegal 1979). The unique *Ae. albopictus* population in which virus was detected has a higher viral load than many other *Ae. aegypti* populations tested suggesting that in some areas, *Ae. albopictus* could intervene in YFV transmission. This result is quite alarming since *Ae. albopictus* has been found most prevalent in some rural [21], urban and peri-urban environments [8–10] in Central Africa. Interestingly, *Ae. albopictus* has been found naturally infected by YFV in Brazil and could serve as bridge vector for transferring enzootic YFV at the urban-forest/rural interface in Central Africa into cities as suggested previously in Brazil [22]. However, other factors should be considered to determine if a mosquito species can act as a vector under natural conditions: mosquito lifespan, trophic preferences or vector abundance [23]. Likewise, bacterial symbionts of mosquitoes have been shown to alter the vector competence to arboviruses [24]; *Ae.* *albopictus* from Brazzaville might have undergone changes in bacteria composition as the 5th generation in the laboratory was used for experimental infections.

Our experiment is the first one establishing the vector competence of *Ae. aegypti* and *Ae. albopictus* towards YFV in Central Africa. We showed that *Ae. albopictus* in a populated city like Brazzaville, can experimentally transmit YFV at 21 dpi suggesting a potential of this species to participate in YFV transmission but perhaps too late to pose an immediate threat for the region. However, YFV can evolve by becoming more adapted for a higher transmission by an unusual vector species [5]. Further studies using a local strain of YFV circulating in Central Africa are needed to validate these results. Our findings support the efforts needed in vector surveillance and control, and vaccine coverage to prevent major YFV outbreak as reported recently in Angola.
